# Stereotactic radiosurgery for recurrent high-grade gliomas: a systematic review

**DOI:** 10.1007/s11060-025-05156-0

**Published:** 2025-07-21

**Authors:** Trent Kite, Bryce Bossinger, Vineetha Yadlapalli, Stephen Jaffee, John Herbst, Stephen Karlovits, Rodney E. Wegner, Matthew J. Shepard

**Affiliations:** 1 Department of Neurosurgery, Allegheny Health Network Neuroscience Institute, Pittsburgh, PA USA; 2 Edward Via College of Osteopathic Medicine, Blacksburg, VA USA; 3 Drexel University College of Medicine, Philadelphia, PA USA; 4 Division of Medical Oncology, Allegheny Health Network Cancer Institute, Pittsburgh, PA USA; 5 Division of Radiation Oncology, Allegheny Health Network Cancer Insitute, Pittsburgh, PA USA

**Keywords:** Stereotactic radiosurgery, High grade glioma, Glioblastoma, Systematic-review

## Abstract

**Purpose:**

Management of recurrent high-grade glioma (rHGG) is challenging. Contemporary therapeutic approaches include systemic chemotherapy, resection, conventional radiation, and stereotactic radiosurgery (SRS). Stereotactic radiosurgery is increasingly utilized given its low toxicity rates and relative efficacy. As the pace of research on this topic is rapidly evolving, a comprehensive review of the existing literature is necessary.

**Methods:**

A systematic review in accordance with the preferred reporting in systematic review and meta-analysis guidelines (PRISMA) was conducted. PubMed and Science Direct databases were queried for articles which reported a primary analysis on a cohort of patients with recurrent gliomas (WHO grade III and IV) treated with SRS. Articles meeting the inclusion criteria and satisfying the quality threshold were included in the final review.

**Results:**

In total 22 articles representing 1,191 patients satisfied the inclusion criteria and quality threshold. The articles spanned a time frame from 1999 to March 2025. Tumor subtypes were distributed as 245 (20.6%) grade III and 946 (79.4%) grade IV. Linear accelerator (LINAC) based SRS was the most frequently utilized SRS platform treating a median tumor volume of 9.9cm^3^ (range: 1.21-44.0) with a median prescription dose of 16.5 Gy. At one-year, the pooled actuarial survival was 53%. At the time of last radiographic follow up, the pooled local progression and distant progression were 58% and 35% respectively. Grade ≥ 3 toxicity ranged from 0 to 14%.

**Conclusions:**

For patients undergoing SRS for rHGG, overall survival times are consistent with alternative salvage therapies (chemotherapy, resection, and conventional radiotherapy) with relatively low treatment-related toxicity. Certain factors such as age, Karnofsky performance status (KPS), WHO grade, and interval between primary tumor treatment and reccurence/salvage SRS may be important in predicting treatment response.

## Introduction

Managing high grade gliomas (HGGs) is one of the most challenging tasks in neuro-oncology [1, 2]. Much of the challenge is derived from the fact that these tumors invariably recur [3]. In the setting of recurrence, there is no widely accepted approach to management [1]. Available therapeutic modalities consist of repeat resection, chemotherapy, and radiation therapy [1]. Many recurrent high-grade glioma (rHGG) patients are not ideal candidates for repeat surgical resection based on performance status [1]. Furthermore, patients with ill-defined tumors in eloquent or deep regions of the brain may not be able to undergo the extent of resection associated with survival improvement as defined by the Response Assessment in Neuro-oncology (RANO) Resect group [3]. Additionally, the stress of surgery in the setting of an aggressive terminal illness may not be an attractive option for many patients [2]. Regardless of individual patient factors, repeat resection is associated with recurrence similar to resection in the primary tumor setting [1]. Chemotherapy, typically consisting of Carmustine, Temozolomide (TMZ) or Prednisone/ Carmustine/Vincristine (PCV) lacks durable efficacy and frequently induces toxic side effects [1, 4]. Further treatment with conventional external beam radiation therapy (EBRT) is accompanied by increased risk of radiation toxicity and necrosis [5].

Much like primary HGGs, previous literature suggests that local tumor control (LC) can potentially improve survival outcomes in the rHGG setting [5, 6]. This is supported by increased disease activity at the margins of the previously treated tumor volume at the time of death in the majority of patients [5]. Given this, it is practical to combine local and systemic therapy in these patients. Stereotactic Radiosurgery (SRS) is delivered via several platforms (Gamma Knife, CyberKnife, and Linear Accelerator (LINAC)), notably exhibiting treatment efficacy on par with that of conventional radiotherapy, with less treatment related toxicity [1]. Additional benefits of SRS include the ability for treatment to be delivered on an outpatient basis and with less costs than open surgical resection [2]. Overall, the primary goal of SRS in the rHGG setting is to improve tumor control while minimizing adverse treatment related events [5]. Consequently, this may translate to an overall survival (OS) and quality of life benefit [6].

To date, there have been a number of retrospective cohort studies examining the role of SRS in rHGGs, however results have been mixed [1]. Given the theoretical benefits of SRS over other available therapeutic modalities further investigation is necessary. In response to this we sought to evaluate the use of SRS in rHGGs (WHO grades III and IV) throughout the literature and provide a comprehensive summary and analysis of the reported outcomes to date.

## Methods

### Inclusion criteria

A systematic review was conducted in accordance with the Preferred Reporting Items in Systematic Reviews and Meta-Analyses (PRISMA) guidelines [7]. Studies including patients with the following characteristics were considered for inclusion in the review: (1) Pathology confirmed primary grade III or IV glioma with tissue diagnosis obtained either from surgery or biopsy at the time of primary intervention (2) Recurrent brain tumor diagnosed radiographically or pathologically in cases where surgery was performed prior to SRS (3) at least 3 months of clinical and radiographic follow up (4) recurrent lesion treated with SRS (i.e. Gamma Knife, LINAC). Patients with primary low-grade gliomas (LGGs) undergoing dedifferentiation into higher grade tumors at the time of tumor recurrence were excluded. Case reports, case series, conference abstracts, review articles, and non-English texts were excluded from the review. The database search was conducted between January of 2025-March 1st, 2025.

### Search strategy

A PubMed and ScienceDirect database search were conducted using the following phrase: ((Glioblastoma OR Recurrent high-grade glioma OR Recurrent GBM) AND (Stereotactic radiosurgery OR Gamma Knife OR Cyberknife OR LINAC)). No date range filter was applied. On the ScienceDirect search a “research article” filter was applied. Results were initially filtered by title concordance with search terms. The remaining articles were screened for relevance based on their abstract. Finally, the remaining articles underwent full text review for relevance and adherence to inclusion criteria. To ensure that no relevant articles were missed during the search a manual review of each article’s references was performed. Two authors (T.K) and (B.B) conduct independent searches, with discrepancies resolved by a third author (V.Y).

### Data extraction

Two authors independently extracted data (T.K) and (B.B). Basic patient information was obtained (age at the time of SRS, WHO grade, chemoradiation details prior to SRS, surgery at the time of SRS for recurrence, chemo at the time of SRS for recurrence). Radiosurgical parameters (tumor volume (cm^3^), maximum dose (Gy), marginal dose (Gy)). Outcome data (time to recurrence or SRS after treatment for primary tumor, time to recurrence after SRS for recurrent lesion, overall survival (OS), progression free survival (PFS) from the time of SRS, chemotherapy after SRS for recurrent lesion, adverse events). The primary endpoints consisted of 1-year OS and tumor control (local and distant) at the time of last radiographic follow up. The following data was input into a central excel sheet and exported for statistical analysis.

### Quality assessment

A Newcastle-Ottawa Scale (NOS) was utilized to assess the strength of the selected studies. Studies scoring > 7 were deemed high quality and included in the final analysis.

### Statistical analysis

Continuous variables were analyzed using descriptive statistics when possible and reported as median (range). The pooled point estimate for our primary endpoints were displayed in respective forest-plots. The method of generating these plots was previously described by Fekete et al. [8]. Briefly a fixed effects model was selected, and inverse-variance weighting method was utilized to aggregate each study input variable into an overall point estimate. For OS, actuarial survival rates as a proportion of the cohort included at the beginning of the study to one year of clinical follow up were aggregated. To compare the effects of chemotherapy on OS, the hazard ratio with a respective 95% confidence interval was extracted from a cox regression analysis. Distant and local progression event rates were obtained by extracting the actuarial estimate given at the last radiographic follow up interval. Using the method described by McGrath et al. an aggregate median OS was calculated for the studies, using the time of SRS as the reference point [9]. In cases where data was not available for aggregate analysis these studies were omitted.

## Results

### Literature review results

**Fig. 1:**
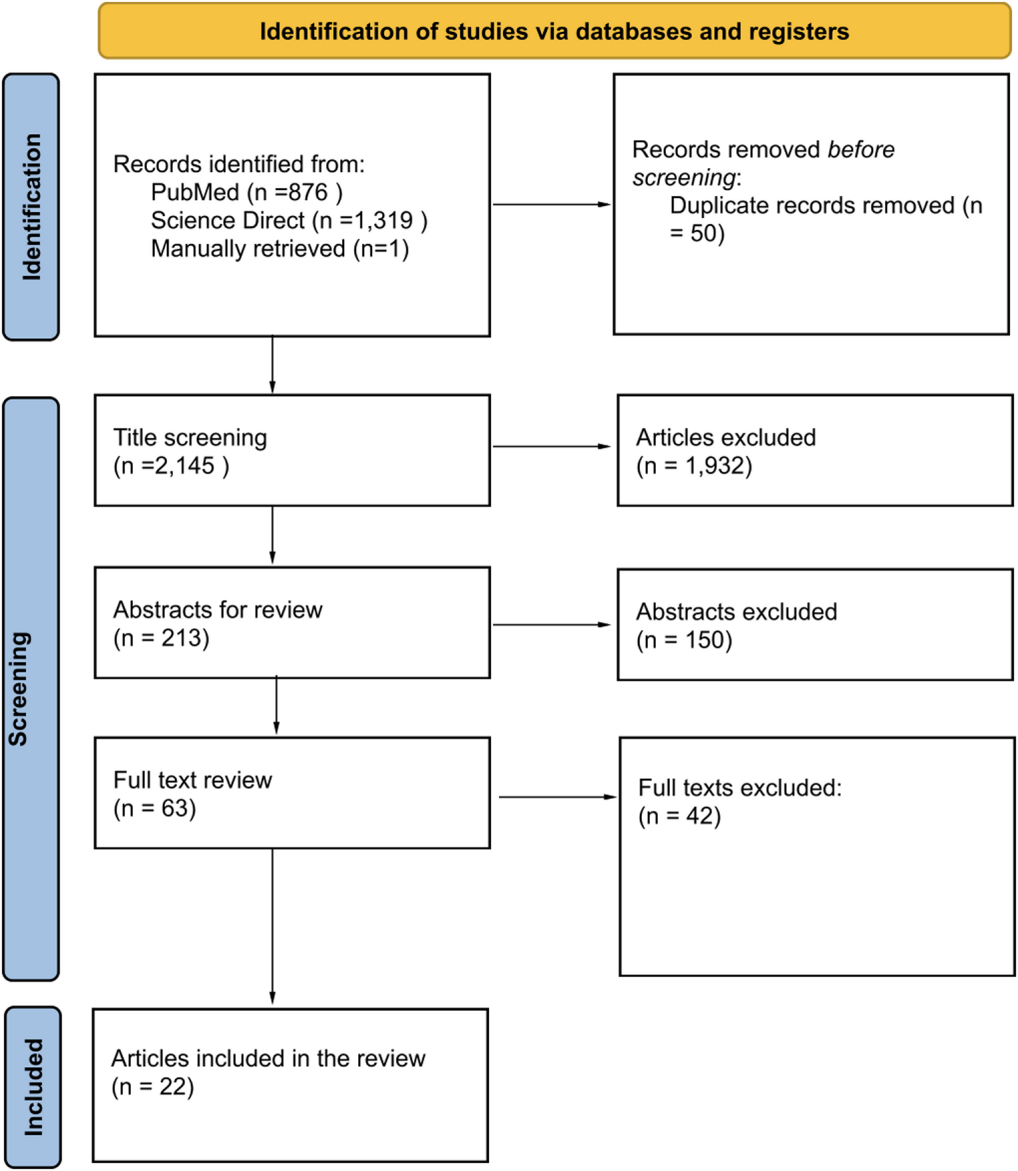
PRISMA flow diagram

An initial database search yielded 2,195 results, with 50 articles removed for duplication resulting in 2,154 articles. From these results 1,932 articles were excluded as their titles did not match the search criteria. Subsequently, 213 abstracts were screened for relevance to the study question and adherence to inclusion criteria. Finally, 63 studies underwent full text review with 21 meeting the inclusion criteria and quality threshold. An additional article was selected manually. Overall, 22 studies reporting on 1,191 patients were selected for review. The selected studies ranged from 1999 to 2025. A PRISMA flow diagram outlining our search process is presented in Fig. 1.

### Study characteristics

The median age of the studies reviewed was 53.0 years (IQR: 50.0–56.0 years). In terms of tumor pathology 245 (20.6%) were grade III and 946 (79.4%) were grade 4. Primary tumor management with concurrent chemoradiation occurred in more than 50% of patients in 18 (100%) of reporting studies. In 13 of the 18 reporting studies 100% of patients received chemoradiation as adjunctive therapy for their primary lesion. The median time to either tumor reccurence or salvage SRS for tumor reccurence was 10.0 months (IQR: 6.9–15.0). In fourteen (70.0%) studies the SRS platform was LINAC based, nine (40.9%) were Gamma Knife, and 1 (4.5%) was an Infini gamma ray. A comprehensive reporting of characteristics by article is provided in Table 1.

**Table 1:** Study characteristics

Study	Total number of patients	Age at SRS (Yrs)	WHO grade (%)	Adjuvant chemoradiation following initial resection (%)	Time to reccurence or SRS following initial resection (months)	SRS platform
Guan et al.	70	53 (20–76)	III: 21 (30.0%) IV: 49 (70.0%)	100.0%	NR	LINAC
Elliot et al.	26	60.4 (36.5–70.6)	III: 10 (38.5%) IV: 16 (61.5%)	100.0%	7.9 (1–11)	Gamma Knife
Imber et al.	174	54.1 (21.8–85.3)	IV: 174 (100.0%)	NR	NR	Gamma Knife
Navarria et al.	25	50 (23–81)	III: 13 (48.0%) IV: 12 (52.0%)	100.0%	19.27 (7–76)	NR
Cho et al.	71	49 (16–75)	III: 29 (59.0%) IV: 42 (41.0%)	62.0%	13.0 (1-228)	LINAC
Koga et al.	9	50 (17–79)	IV: 9 (100.0%)	100.0%	6.0 (1–10)	Gamma Knife
Kong et al.	114	49 (5–75)	III: 49 (43.0%) IV: 65 (57.0%)	100.0%	Grade III: 11.0 (2.0–68.0) Grade IV: 4.3 (1.5–27.0)	LINAC Gamma Knife
Maranzano et al.	22	55 (27–81)	IV: 22 (100.0%)	100.0%	9 (5–25)	LINAC
Combs et al.	32	56 (33–76)	IV: 32 (100.0%)	NR	10.0 (1–77)	LINAC
Vordermark et al.	19	50 (11–74)	III: 5 (36.0%) IV: 14 (64.0%)	100.0%	4.9 (1.3–37.3)	LINAC
Fogh et al.	147	53 (19–86)	III: 42 (29.0%) IV: 105 (71.0%)	74.8%	8 (4-205)	NR
Sirin et al.	19	47 (23–65)	IV: 19 (100.0%)	57.9%	9 (5–49)	LINAC
Yazici et al.	37	37 (22–68)	IV: 37 (100.0%)	100.0%	NR	LINAC
Gigliotti et al.	25	54 (23–74)	III: 5 (20.0%) IV: 20 (80.0%)	100.0%	18 (3–71)	LINAC
Reynaud et al.	32	57.5 (29–76)	III: 14 (43.7%) IV: 18 (56.3%)	81.3%	1.3 (0-8.4)	LINAC
Sadik et al.	49	NR	NR	NR	NR	Gamma Knife
Guseynova et al.	126	56 (17–80)	IV: 126 (100.0%)	100.0%	12 (1–96)	Gamma Knife
Lovo et al.	46	50.3 (19–81)	IV: 46 (100.0%)	100.0%	NR	LINAC Infini Gamma Ray
Park et al.	23	53 (36–80)	IV: 23 (100.0%)	65.0%	NR	LINAC Gamma Knife
Frischer et al.	42	57.2 (15.7–81.9)	IV: 42 (100.0%)	100.0%	17.0 (3.9–57.9)	Gamma Knife
Sallabanda et al.	50	51.5 (21–81)	III: 26 (52.0%) IV: 24 (48.0%)	NR	23.2 (1-196)	LINAC
Kite et al.	33	60 (51.5–68.0)	III: 6 (18.0%) IV: 27 (82.0%)	100.0%	12 (9.0-18.5)	Gamma Knife

Abbreviations: SRS: stereotactic radiosurgery, WHO: world health organization, NR: not reported, LINAC: linear accelerator

### Treatment parameters

The median tumor volume at SRS was 9.9 cm^3^ (IQR:5.3–20.7). Recurrent lesions were treated with a median prescription dose of 16.5 Gy (14.7–26.2) (Table 2). Overall, 11 (50%) studies utilizing single fraction SRS, 5 (22.7%) fractionated SRS, and 6 (27.3%) both single and fractionated SRS. Eight studies reported fractionation schemes with a median number of treatment fractions of 5 (range: 2–20). The use of chemoradiotherapy at the time of SRS ranged from 0 to 86.4%. The most frequently used adjuvant chemotherapy across reporting studies for reccurence was TMZ (45.4%) followed by BVZ (40.9%). Reported Grade 3 toxicity across 15 reported studies ranged from 0 to 14%. Reported radiation necrosis rates ranged from 0 to 36% over 17 reporting studies.

**Table 2:** Treatment deatils for patients undergoing SRS

Study	Tumor Volume (cm^3^)	Prescription Dose (Gy)	Single or Fractionated	Surgery for Reccurence (%)	Chemotherapy for Reccurence (%)	Chemotherapy Regimen (*N*)
Guan et al.	16.8 (0.81-121.96)	24 (12–30)	fractionated (2–6 fx)	NR	100%	TMZ BVZ TMZ + BVZ BSC
Elliot et al.	1.21 (0.27–11.9)	NR	Single	NR	NR	NR
Imber et al.	7.0 (0.3–39.0)	16 (10–22)	Single	NR	44%	TMZ CCNU BCNU Thalidomide BVZ
Navarria et al.	35.0 (2.46–116.7%)	25	Fractionated (5–10 fx)	44.0%	86.4%	TMZ BVZ ACNU Fotemustine PC Scheme
Cho et al.	14.0 (1-115)	SF (17, 9–40) FSRS (37.5, 20–45)	Single/Fractionated (10–20 fx)	15.5%	NR	NR
Koga et al.	NR	20	Single	77.7%	NR	NR
Kong et al.	10.6 (0.09–79.6)	16 (12–50)	Single	NR	NR	NR
Maranzano et al.	44.0 (1.4–151)	30	Single/Fractionated (NR)	100%	7.3%	TMZ BVZ
Combs et al.	10.0 (1.2–59.2)	15 (10–20)	Single fraction	NR	NR	NR
Vordermark et al.	15.0 (4–70)	SF (5, 4–10) FSRS (30, 20–30)	Fractionated (2–6 fx)	26.3%	68.4%	TMZ ACNU VM-26 Other
Fogh et al.	22.0 (0.6–104)	NR	Fractionated (NR)	57.0%	32.7%	TMZ TMZ/BVZ Irinotecan/Epithilnone/Sunitinib/Bortezomib/Sorafenib/VincristineBVZ/Irinotecan
Sirin et al.	13.0 (7.0–19.0)	16 (10–19)	Single	0%	0%	NR
Yazici et al.	24.0 (2–81)	30 (14-32.5)	Single/Fractionated (1–5 fx)	NR	NR	NR
Gigliotti et al.	9.8 (0.55–92.78)	25 (16-27.5)	Single fraction (1–5 fx)	28%	12%	TMZ/Veliparib TMZ/BVZ BVZ
Reynaud et al.	6.1 (0.1–42.2)	30 (27–30)	Fractionated (5–9 fx)	21.88%	52.65%	PCV/TMZ/BVZ/Lomustine/Fotemustine
Sadik et al.	NR	NR	Single	NR	NR	NR
Guseynova et al.	3.75 (0.04–37.10)	12 (10–25)	Single	NR	NR	NR
Lovo et al.	Cone based (5.6, 0.2–65.5) Infini (3.2, 0.1–64.8) Cyberknife (36.9, 6–71)	Cone based (14, 8–20) Infini (15, 10–24) Cyberknife (16, 15–17)	Single/Fractionated (NR)	NR	32.6%	BVZ TMZ
Park et al.	9.9 (0.8–37.9)	15 (12–20)	Single	NR	NR	NR
Frischer et al.	5.1 (0.6–15.0)	10 (6–16)	Single	7.14%	83.0%	NR
Sallabanda et al.	NR	14 (8–20)	Single	NR	NR	NR
Kite et al.	5.2 (0.6–13.1)	25.0 (25.0-29.3)	Single/Fractionated (3–5)	0%	39.4%	TMZ BVZ

### Outcomes

**Fig. 2:**
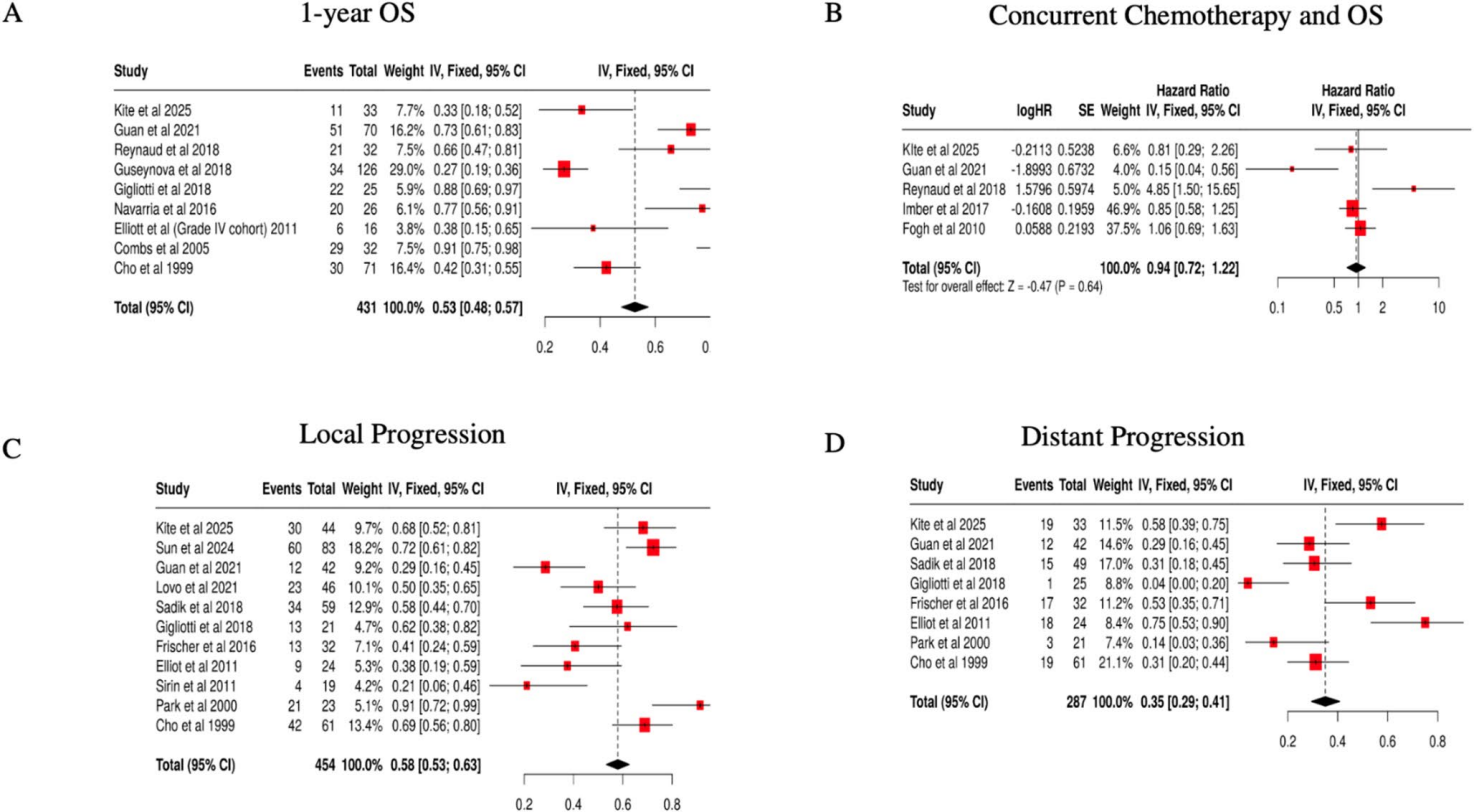
Pooled analysis of 1-year OS, concurrent chemotherapy and SRS on survival, local and distant progression rates (**a**) pooled 1-year actuarial survival (**b**) pooled hazard ratios for the effect of concurrent chemotherapy and SRS on overall survival (**c**) pooled local progression (**d**) pooled distant progression

The pooled one-year actual survival was 53%. (95% CI: 0.48–0.57) (Fig. 2A). When examining studies which analyzed the effects of chemotherapy on OS in a regression analysis the pooled hazard ratio was 0.94 (95% CI: 0.72–1.22) (Fig. 2B). Finally, the pooled actuarial local progression and distant progression at the last clinical follow up were 58% (95% CI:0.53–0.63) and 35% (95% CI:0.29–0.41) respectively (Fig. 2C/2D). The median survival from the time of SRS across all 22 studies is 10.6 months (range: 1.0-223.0).

## Discussion

### Summary of findings

Overall, the analyzed cohort was comprised of mostly WHO grade IV patients. In most of the studies the entire cohort received adjunctive chemoradiation at the time of initial resection. Five studies did not report 100% adjuvant chemotherapy rate, with one of them published prior to 2005 and the adoption of the Stupp protocol, and the other four not providing any further details. However, all patients included in these studies received EBRT following initial resection. At the time of reccurence the median prescription dose across the studies was surprisingly low. Few studies reported any grade ≥ 3 radiation toxicity or radiation necrosis. At one year of clinical follow slightly more than half of the patients in the pooled analysis were alive. Consistent with previous literature local control was more frequent than distant control, however distant control was demonstrated in greater than a quarter of patients in the pooled analysis. This highlights the importance distant control in rHGGs, emphasizing the role of systemic therapy in this setting. Unfortunately, data on the chemotherapy protocols was limited across the studies.

### Survival

Identifying prognostic factors and intervention protocols to prolong survival in patients with rHGG is a persistent challenge. The current estimate of overall median survival following SRS for rHGG is 10.6 months as demonstrated in our analysis. Comparatively surgery and chemotherapy for rHGG demonstrate overall survival of 3–13 months and 5–6 months respectively [10–20]. A frequently observed predictor of improved survival in patients with rHGG undergoing SRS is the interval between surgery for the primary tumor reccurence [4, 21]. This finding is perhaps a reflection of more indolent biology. Imber et al. defined an interval of 20.2 months from the time of surgery to SRS as significantly associated with improved survival [22]. A more conservative estimate was reported by Lovo et al. with a survival benefit for patients undergoing SRS ≥ 10 months from surgery [21]. Given that most recurrences occur prior to 20 months the findings by this study are more applicable. Performance status as measured by the Karnofsky Performance Status (KPS) consistently predicts survival in this patient population. However, the threshold at which KPS predicts improved survival is variable with reports ranging from KPS > 70 to KPS > 90 [4]. Age at the time of SRS is also reported to be associated with survival outcomes [2, 15]. Increasing age, which may be related to declining performance status is invariably associated with diminished survival. Tumors designated as WHO grade III have a relatively prolonged survival compared to grade IV tumors, which may be attributable to more aggressive growth dynamics of grade 4 tumors [23]. Finally, tumor volume at the time of SRS may predict survival with increased tumor volume at the time of SRS associated with diminished survival. Overall, younger age, higher KPS, and smaller tumor volume are persistently associated with improved survival outcomes. Age demonstrated a strong relationship as studies with ≥ 70% survival at 1-year reported a median age of < 60 years across each of these studies.

### Radiosurgical parameters

The use of single fraction over hypofractionated SRS, volumetric constraints, dose escalation, and tumor location have been described as primary factors influencing response to SRS in rHGG [15, 24–26]. Presently there is no clear evidence supporting the use of a single session fraction SRS versus fractionated SRS (fSRS). Fractionation has theoretical radiobiological advantages over single session SRS. Administering SRS over multiple sessions allows for larger tumor volumes to be treated with higher radiation doses [15, 24, 27]. Also, tumors localized to eloquent regions or deep in the skull base may be more amenable to SRS with a fractionated schedule given the need to avoid large doses to critical structures [25]. As the size of the tumor volume at SRS is associated with survival outcomes, it is important to consider that size may have historically limited delivery of appropriate radiation doses to larger tumor volumes [10]. A wide range of volumetric cutoffs have been proposed from < 14cm^3^ to 24cm^3^ [26, 28]. Vordermark et al. reported that larger prescribed doses (> 15 Gy) up to 30 Gy were associated with improved survival [29]. Reynaud et al. escalated the dose to a mean of 35 Gy demonstrating a survival benefit [30]. In contrast Fogh et al. reported increased radiation toxicity with doses over 40 Gy which may act as an upper bound for the therapeutic window of radiation [25]. Furthermore, in the context of a propensity for local reccurence, investigation of the parameters applied to the tumor margin has been conducted. Guan et al. argued that the improved tumor control rate in their cohort was partially a result of delivering radiation to the margins with an isodose line of 63–75% resulting in higher overall marginal irradiation [26]. Consequently, debate over how to best define the clinical target volume (CTV) exists, and is made difficult in the recurrent setting as radiographic appearance of the tumor may be impacted by surgical/radiation effects the primary tumor treatment [31]. Bell et al. suggested that poor delineation of the clinical target volume was responsible for underdosing of the recurrent target volume [31]. In their work the authors demonstrated the role of the emerging whole brain spectroscopy technology [31]. Through the use of a Choline: N-acetylaspartate ratio > 2 representing disease activity, a clinical tumor volume of 2 cm resulted in total disease coverage approaching 99%, while no CTV expansion resulted in only 54% disease coverage [31]. Indeed, the authors of this study highlight the need to properly account for microscopic disease in the recurrent setting through the use of CTV expansion.

### Systemic therapy

Chemotherapy has been a mainstay in the management of rHGG patients yet is limited by heterogeneity in reported clinical outcomes. While TMZ has historically been a backbone of systemic therapy, novel agents directed at more specific glioma properties have been developed [10]. A contemporary therapy under intense investigation is the anti-vascular endothelial growth factor (VEGF) monoclonal antibody Bevacizumab (BVZ). Previous studies have demonstrated that BVZ in combination with SRS potentially limits radiation toxicity, namely radiation necrosis [4, 22]. However, the role of BVZ in combination with SRS is variably associated with survival outcomes with many studies reporting no significant improvement in overall survival [4]. While more work is to be done to validate the role of BVZ in improving survival for rHGG patients undergoing SRS, there may be a role in radiation associated toxicity reduction. A methodological limitation of the current literature reporting on outcomes in patients receiving multiple lines of chemotherapy, particularly investigational therapies is a trend toward selection bias. Many of the patients in these studies demonstrate higher performance status, which may confound outcomes [29].

### Tumor control

Reccurence proximal to the treatment volume is a central issue in both primary and rHGG. Most patients in both disease settings experience recurrent disease within a few millimeters of the treatment volume [3]. This pattern of failure is referred to as local progression [14]. A primary barrier to successful repeat resection in rHGG is the diffuse nature of growth resulting in poorly defined tumor margins [30, 31]. In part, the inability to clearly define resectable tumor may result in residual tumor growth adjacent to the resection cavity. Similarly, radiation planning may be limited in the same way when attempting to design a conformable plan to ambiguous tumor margins. Furthermore, as SRS is defined by a steep dose gradient, a portion of the dose may fail to capture marginally invading tumor cells [30–32]. One solution has been the introduction of extended field radiation [32]. Koga et al. demonstrated improved local control using an extended field radiation approach over conventional SRS, albeit without a significant survival benefit [33]. This approach may be promising however, larger lesions (> 20 mm) may not be eligible because an expanded radition field of such magnitude may place the adjacent uninvolved parenchyma at excessive toxicity risk [33]. However, this study did not report a significant increase in radiation toxicity in the extended field SRS cohort compared to the conventional SRS cohort [33]. Overall, the use of a so-called clinical target volume which accounts for additional treatment area outside of the enhancing lesion has been inconsistently utilized in this setting and warrants further exploration [5].

### Adverse events

The typical adverse (AEs) noted in SRS treatment of rHGG are low grade (Grade 1) non-specific acute toxicity such as: alopecia, skin erythema, headaches, and nausea/vomiting [34]. Multiple radiation treatments over a lifespan elevate the risk of radiation induced toxicity, tissue damage, or secondary malignancy [22]. Given this, there ought to be attention to the interval between the radiation administered for the primary tumor and the recurrent tumor. In fact, Imber et al. demonstrated in their cohort of rHGGs an elevated risk of SRS related AEs in patients with a shorter interval from primary radiation treatment [22]. Additionally, the authors of this study demonstrated higher rates of radiation necrosis with treatment of larger tumor volumes [22]. These findings may be related to the associated dose escalation with large tumor volumes [22]. Large tumor volumes requiring increased dose of radiation have increasingly benefited from the adoption of hypofractionated shchemes which allow for similar biologically equivalent dose application as single session schemes with reduced adverse radiation events [25, 26]. It is also important to discriminate clinically relevant radiation necrosis from other instances. Most importantly, cases of radiation necrosis necessitating surgical intervention represent the most clinically significant cases and should be reported separately in future work. Finally, the assessment of radiation necrosis is evolving and has increasingly incorporated new diagnostic modalities like magnetic resonance spectroscopy (MRS) and single photon emission computed tomography (SPECT) [34]. Perhaps the evolution of these technologies will allow for a more accurate delineation of RN allowing for the development of consistent assement and reporting standards Alternatively, the authors of this study demonstrated an inverse relationship between radiation necrosis and time between primary irradiation and SRS [22]. Cumulative radiation exposure is a recognized risk factor for toxicity, and a patient’s history of exposure ought to be accounted for when selecting for further radiotherapeutic treatment [22]. Ultimately these findings provide support for the argument favoring fSRS in larger tumor volumes as discussed previously. There may be additional benefit from redefining how radiation necrosis in these cohorts is presented. Not all cases of radiation necrosis are clinically equivalent, and the most significant cases are those requiring additional surgery. Therefore, modifying radiation necrosis with the need for surgical intervention could contribute to more meaningful data reporting [10].

### Limitations

As with any systematic review the strength of the analysis reflects the individual studies included in the analysis. One of the persistent limiting factors present in most of the studies included in our analysis was the heterogeneity with respect to WHO grade. Many of these studies included both grade III and IV tumors. Higher grade tumors have consistently been associated with diminished overall survival. Given this it is important to understand that the overall survival point estimate may be an overestimate. Furthermore, our review includes patients spanning multiple iterations of WHO grading which means there are potential misclassifications of tumors in older studies relative to more recently published studies. For example, the WHO update in 2016 and 2021 may not be reflected in articles published prior to these years. Similarly, our analysis includes studies published prior to 2005 and the widespread adoption of the Stupp protocol for the management of primary Glioblastoma [36]. Studies published prior to 2005 in our analysis on average may have relatively less overall survival. Furthermore, many of the papers included in the analysis reported varying degrees of primary tumor resection. Additionally, a clear definition of gross total resection and subtotal resection was not consistently reported. As a result, this may have contributed to wide range of SRS treatment volumes, and differential growth patterns introducing excess variability across SRS planning [23]. Furthermore, the classification of “recurrent disease” is dubious, while standardized reporting of reccurence using the RANO-glioma criteria has increased in utilization, some of the earlier papers in this review did not incorporate this criteria. Another limitation included inconsistent reporting of molecular markers associated with survival including but not limited to: MGMT methylation, IDH mutation, CDK2NA homologous deletion, 1pq19 deletion, TERT and EGFR promoter mutations.

Finally, there are a number of key limitations which ought to be considered for future work. Most importantly there is a pervasive issue in the current literature as it pertains to consistent reporting of data. This is highlighted best by the absence of specific surgical as well as chemotherapy deatils across many of the studies summarized herein. Chemotherapy undoubtedly plays an important role in the recurrent setting. While the exact details of which chemotherapy regimens are most efficacious it is important that any study reporting on outcomes in this patient population make an effort to include granular details on the exact regimens employed by the study investigators. Furthermore, we believe it is imperative to define the interval between the completion of adjuvant treatment for the primary tumor to the time of first recurrence as this represents a treatment free interval and is most meaningful to patients. Many studies analyzed time from surgery to recurrence, or time from initial diagnosis to recurrence, which are important parameters but may lack relevance to patients. Moreover, some tumors may display aggressive biology, relating to a “rapidly progressive” phenotype. Consistently defining an interval by which studies can identify such characteristics may contribute to individualized follow-up and management.

### Future research

To date the management of rHGG is largely individualized and lacks widespread consensus. Consequently, there is a need to further define outcomes for a variety of treatment modalities in this setting. Ultimately, support for a standard treatment protocol should be gained through prospective comparative analysis. Examples of highly impactful studies would include single fraction SRS versus fractionated SRS, and SRS with and without concurrent therapy and resection with and without SRS to the post-operative cavity to name a few. Furthermore, tumor treating fields (TTF) is an emerging technology in this space and has been previously been studied in the rHGG setting [35–42]. When combined with chemotherapy, TTF has demonstrated prolonged survival with minimal toxicity, however, there are substantial concerns regarding patient compliance [35–42]. It is imperative to limit treatment related toxicity in this patient population, and therefore TTF represents a viable alternative to SRS and therefore the literature would benefit from direct comparative analysis. To date a trend to towards success with SRS in rHGG with young patients, good performance status, and small tumor size has been reported [42–50]. Based on these findings, a simple nomogram incorporating these factors could prove meaningful in supporting the decision to proceed with SRS over alternative therapies. It is clear that WHO grade mediates survival outcomes; therefore, analyzing outcomes for grade III and IV tumors separately may be appropriate. Given the recent WHO update in 2021 [51], with the recognition of isocitrate dehydrogenase (IDH) mutant status, examination of IDH-wildtype and IDH-mutant pathology treated with SRS in the recurrent setting will reveal potential differences in radio responsiveness of these respective subtypes. To our knowledge this has yet to be examined in a comparative study, however as molecular signatures have been established as critical prognostic factors this may have introduced bias in studies reporting prior to 2021.

While local control is a primary endpoint for this population, distant recurence can complicate management even further. Other than Kite et al., there were no studies conducting a regression analysis of local tumor control following SRS. Further there were no studies reporting a regression analysis for distant tumor control following SRS. Indeed, in their analysis Kite et al. demonstrated a non-significant relationship between BVZ at the time of SRS on local tumor control (HR: 0.90 *p* = 0.81) or any chemotherapy following SRS (HR:0.71, *p* = 0.50 [52]. This analysis should be extended to distant tumor control as chemotherapy is likely the most influential in this aspect of disease control. Understanding the influence of a multifocal disease process on SRS candidacy deserves further investigation. Finally, an investigation of molecular signatures associated with improved radiotherapy response may better inform the decision to proceed with SRS in select cases. Previously, MGMT promoter methylation was demonstrated to portend improved OS, which may influence the selection of chemotherapy at the time of SRS [52].

## Conclusion

Management of rHGG is variable and lacks robust clinical evidence. Historically, options were limited to repeat resection and chemotherapy. However, radiation therapy, particularly stereotactic radiosurgery has emerged as a less invasive and relatively safer alternative. To date there have been few attempts in the literature to comprehensively review this topic and provide an estimate of overall survival and tumor control. Herein we report a median overall survival similar to repeat resection but greater than chemotherapy alone. However, local and distant progression following SRS emphasize the need for adjunctive therapy and the systemic nature of the disease process.

## Data Availability

Data available upon reasonable request.
